# Letrozole-induced endometrial preparation improved the pregnancy outcomes after frozen blastocyst transfer compared to the natural cycle: a retrospective cohort study

**DOI:** 10.1186/s12884-022-05174-0

**Published:** 2022-11-07

**Authors:** Kenji Ezoe, Junichiro Fukuda, Kazumi Takeshima, Kazunori Shinohara, Keiichi Kato

**Affiliations:** Kato Ladies Clinic, 7-20-3 Nishishinjuku, Shinjuku-Ku, Tokyo, 160-0023 Japan

**Keywords:** Blastocyst transfer, Congenital anomalies, Live birth, Pregnancy complications, Propensity score

## Abstract

**Background:**

Letrozole treatment is considered an effective option in endometrial preparation for frozen embryo transfers in patients with ovulation disorders or irregular menstruation; however, the effectiveness of letrozole-induced endometrial preparation remains unclear in ovulatory patients. Furthermore, there is no comparative study reporting on pregnancy complications and congenital anomalies after frozen embryo transfers comparing natural and letrozole-assisted cycles. This study examined whether letrozole-induced endometrial preparation affected pregnancy outcomes, perinatal outcomes, and congenital anomalies after single vitrified-warmed blastocyst transfers (SVBTs) in ovulatory patients, as compared with the natural cycle.

**Methods:**

This historic cohort study included only patients with unexplained infertility. Overall, 14,611 patients who underwent SVBTs between July 2015 and June 2020, comprising both natural and letrozole-assisted cycles, were included. Multiple covariates that impact outcomes were used for propensity score matching; 1,911 patients in the letrozole group were matched to 12,700 patients in the natural group, and the clinical records of 1,910 patients in each group were retrospectively analysed. Cycle characteristics, pregnancy outcomes (clinical pregnancy, ongoing pregnancy, and live birth), and incidence of pregnancy complications and congenital anomalies were statistically compared between the two groups.

**Results:**

Multivariate logistic regression analysis showed that letrozole administration during SVBT cycles significantly improved the live birth rate (*P* = 0.0355). Gestational age, birth length, birth weight, and infant sex, as well as the incidence of pregnancy complications and birth defects, were statistically comparable between the two groups. Furthermore, multivariate logistic regression analysis revealed that the perinatal outcomes were not affected by letrozole-induced endometrial preparation.

**Conclusions:**

Letrozole-induced endometrial preparation improved the live birth rate compared with the natural cycle, without adverse effects on perinatal outcomes and congenital anomalies after SVBTs. Therefore, letrozole-induced endometrial preparation might be a safe and more effective strategy, especially for patients with insufficient luteal function.

**Supplementary Information:**

The online version contains supplementary material available at 10.1186/s12884-022-05174-0.

## Background

Letrozole belongs to the type-I class of non-steroidal aromatase inhibitors that bind competitively to CYP19A1 [1]. Follicular development can be induced by the administration of letrozole via suppressing androgen conversion into oestradiol and increasing serum gonadotropin-releasing hormone levels [2]. Therefore, letrozole is considered effective for ovulation induction and increased follicle recruitment in cases of ovulatory infertility [3]. The efficacy of letrozole administration for controlled ovarian stimulation was also reported in multiple subfertile and infertile populations, including patients with a poor ovarian response [4–7], polycystic ovarian syndrome [8–11], and unexplained infertility [12].

Recently, letrozole has also been used for endometrial preparation in frozen embryo transfer (FET) cycles [13–16]. The endometrial preparation with letrozole resulted in physiologic serum oestradiol levels and more favourable endometrial morphology, compared to ovarian stimulation cycles [17, 18]. Letrozole-induced cycles had improved clinical pregnancy and live birth rates after FETs, compared to artificial and gonadotropin-based ovarian stimulation cycles [14–16]. Therefore, letrozole treatment is considered an effective option in endometrial preparation for FET in patients with ovulation disorders or irregular menstruation. Conversely, in ovulatory patients, the effectiveness of letrozole-induced endometrial preparation remains unclear since the studies comparing pregnancy outcomes after FETs between natural and letrozole cycles are limited. Some studies have reported that the pregnancy outcomes after frozen cleaved embryo transfers (ETs) were comparable between the two protocols [13, 14]. In a recent study, propensity score matching was performed to balance the baseline characteristics between two cohorts; the study confirmed that endometrial preparation with letrozole could achieve similar pregnancy outcomes after frozen cleaved ETs when compared with the natural cycle [19], suggesting that letrozole-induced endometrial preparation might have some benefit after frozen cleaved ET cycles. Currently, only one study has compared pregnancy outcomes after frozen blastocyst transfers (FBTs) between the natural and letrozole-induced cycles. This study reported that in the letrozole group, the rates of clinical pregnancy and live birth after FBTs were improved, compared with those of the natural group [20]. However, in that study, the characteristics of patients included in the natural and letrozole groups were remarkably different; factors potentially affecting pregnancy outcomes, such as developmental speed and morphology of blastocysts, were not included as confounders in their multivariate analysis. Therefore, the effectiveness of letrozole-induced endometrial preparation in FBT cycles remains unclear and should be evaluated among patients with similar baseline characteristics. Furthermore, there is no comparative study reporting on pregnancy complications and congenital anomalies after FETs between the natural and letrozole-induced cycles. At our centre, letrozole-induced endometrial preparation is occasionally used for patients with normal ovulation. As we have experienced that endometrial preparation using letrozole improves the luteal function, letrozole-induced endometrial preparation is considered an effective strategy, especially for patients with unexplained infertility who exhibit luteal disfunction. In the present study, to investigate whether the letrozole-induced endometrial preparation was effective for patients with unexplained infertility, letrozole-induced cycle pregnancy outcomes, after single vitrified-warmed blastocyst transfers (SVBTs), were compared to those of natural cycles in patients with normal ovulation, using propensity score matching. Furthermore, to examine whether letrozole administration had adverse effects on maternal and infant health, the maternal and perinatal outcomes were also compared between the two protocols for endometrial preparation.

## Methods

### Study patients

Clinical records of women who underwent natural and letrozole-induced cycles during their first SVBTs at the Kato Ladies Clinic between July 2015 and June 2020 were retrospectively analysed. The oocytes of each patient were used during treatment. This study only included one cycle per patient diagnosed with unexplained infertility. To homogenise patient characteristics, patients with anovulatory infertility, oviductal infertility, male infertility, and endometrial infertility were excluded from this study. Patients with recurrent implantation failure [four or more unsuccessful ETs [21] were also excluded.

### Minimal ovarian stimulation cycle in vitro fertilisation

In the present study, for the oocyte retrievals, the ovarian stimulation used was only clomiphene citrate (CC) based minimal stimulation. The detailed protocol for minimal stimulation with CC has been previously reported [22–24]. Briefly, CC (50–100 mg/day; Fuji Pharma Co., Ltd., Tokyo, Japan) was orally administered from the 3rd day of the retrieval cycle to the day before induction of final oocyte maturation. Ovulation was triggered using a nasal spray containing the gonadotropin-releasing hormone agonist, buserelin (300 μg: Suprecur; Mochida Pharmaceutical Co., Ltd., Tokyo, Japan or Buserecur; Fuji Pharma Co., Ltd.).

Oocyte retrieval was performed at 34–36 h after triggering, using a 21-G needle (Kitazato Corporation, Shizuoka, Japan) without anaesthesia or follicular flushing. Cumulus-oocyte complexes were collected, washed, and transferred to human tubal fluid medium (Kitazato Corporation) with paraffin oil at 5% CO_2_ in air at 37 °C for culturing, until either conventional in vitro fertilisation was performed 3 h later [25] or in cases of intracytoplasmic sperm injection, denudation was performed at 4 h after oocyte retrieval [26]. All embryos were cultured at 37 °C (gas phase: 5% O_2_, 5% CO_2_, and 90% N_2_) with 100% humidity in a water jacket or with non-humidified incubators (Astec Co. Ltd, Fukuoka, Japan). Embryo warming was performed using Cryotop® (Kitazato Corporation), as described previously [27, 28].

### SVBT in natural and letrozole cycles

We have experienced that the endometrial preparation using letrozole improves the luteal function. After the explanation of this benefit, the endometrial preparation method was determined in consultation with patients; their preferences were often considered. In the natural cycle group, the only pharmacological intervention was the administration of a gonadotropin-releasing hormone agonist for the induction of final oocyte maturation and ovulation [23, 29]. Monitoring included transvaginal ultrasonography and blood hormone (oestradiol and progesterone) testing performed on days 5–21, according to the patient’s cycle length. When the leading follicle reached 18 mm in diameter, ovulation was triggered using buserelin. In the letrozole cycle group, letrozole (Novartis, Basel, Switzerland, or Fuji Pharma Co., Ltd.) was administered at a dosage of 2.5 mg/day on days 3–7, and follicular development was monitored via hormone assay and ultrasonography [30]. If follicular development was unsuccessful in this manner, letrozole was administered for 6 days starting on day 4 of menstruation, at a starting dose of 7.5 mg/day, and tapered off. When the leading follicle reached 18 mm in diameter, ovulation was triggered using buserelin.

The serum oestradiol and progesterone levels were measured in the morning of SVBT. SVBT was performed as previously described [26, 31]. The ET procedure was performed under vaginal ultrasound guidance using a specially designed soft silicone inner catheter (Kitazato Corporation) by inserting a single embryo at a minimal volume into the upper part of the uterine cavity. Dydrogesterone (30 mg/day) was administered orally during the early luteal phase after SVBT. In addition, cases with insufficient luteal function (progesterone level on the day of SVBT < 11 ng/mL), progesterone was administered intravaginally (Lutinus, Ferring Pharmaceuticals, Saint Prex, Switzerland) until the 9^th^ week of pregnancy. Implantation was defined by the serum human chorionic gonadotropin level (> 20 mIU/mL), in accordance with a previous study [32]. The clinical and ongoing pregnancy rates were defined according to the ultrasonographic observation of a gestational sac, 3 weeks after SVBTs, and the observation of a foetal heartbeat 5 weeks after SVBTs, respectively [31]. Live birth was defined as delivery at ≥ 22 weeks of pregnancy. Early pregnancy loss and miscarriage were defined according to the absence of a gestational sac after implantation as well as the absence of live birth after the confirmation of a gestational sac.

### Study outcomes

The primary outcome was the live birth rate after SVBTs. The secondary outcomes were perinatal complications and major anomalies. Perinatal complications included pregnancy complications (hypertensive disorders of pregnancy; gestational diabetes mellitus; haemolysis, elevated liver enzymes, and low platelet count syndrome; preterm premature rupture of membrane; low-lying placenta; placenta previa; placenta accreta; placental abruption; and caesarean section) and neonatal outcomes (gestational age, birth weight, small for gestational age [SGA], and large for gestational age [LGA]).

A questionnaire was utilised, requesting information regarding the following: date and mode of delivery, sex, birth weight, and length of the newborn(s); presence of any birth defect or other anomaly; and pregnancy complications [33]. Preterm delivery was defined as delivery occurring at < 37 weeks [34]. Low birth weight was defined as a birth weight < 2,500 g. SGA and LGA were defined as birth weight below the 10^th^ percentile and above the 90^th^ percentile, respectively, according to the Japanese national reference for neonates [35]. Neonatal outcomes were obtained from questionnaires completed by mothers after their 1-month infant examination. Birth defects were classified using the Q-codes of the International Statistical Classification of Diseases and Related Health Problems, 10^th^ Revision, with classification being performed by reformatting the answers to the parent questionnaires [36].

### Statistical analyses

In the present study, propensity score matching was performed using JMP software (SAS, Cary, NC, USA) to reduce the bias in patient characteristics, as previously reported [22]. All statistical analyses were performed using JMP software. Proportion data were analysed using the chi-squared test. Continuous parameters were compared using Student’s *t*-test. Univariate logistic regression analysis was performed to identify confounders that were potentially associated with pregnancy, maternal, and perinatal outcomes (Supplementary Table 1). The likelihood ratio test for the significance of the coefficient was performed, and variables with *P* < 0.1000 were used as confounders. Similarly, multivariate logistic regression analysis for the pregnancy, maternal, and perinatal outcomes was performed to adjust the bias (using the confounders) and to verify the statistical significance (using Wald statistic). Odds ratios (ORs) and adjusted ORs (AORs) are reported with 95% confidence intervals (CIs) for each group. The natural group was used as the reference for the logistic regression analysis. The calculation of statistical power and sample size was performed using G*Power (Heinrich-Heine-Universität Düsseldorf, Düsseldorf, Germany). Statistical significance was set at *P* < 0.05.

## Results

### Characteristics of the study cohort

In this study, the blastocysts produced via CC-based minimal stimulation were used. The embryonic outcomes were comparable between the natural and letrozole groups (Supplementary Table 2). A total of 14,611 SVBTs (natural, 12,700 cycles; letrozole, 1,911 cycles) were performed during the study period (Table 1, Supplementary Fig. 1). Before propensity score matching, women and men in the natural group were significantly older than those in the letrozole group (*P* < 0.0001). The body mass index, number of previous oocyte retrievals, number of previous ETs, previous caesarean section, and previous smoking were comparable between the groups. However, the proportion of previous deliveries and previous miscarriages was significantly different between the groups. Furthermore, the quality of transferred blastocysts, including culture time to the expanded blastocyst stage (*P* < 0.0001), blastocyst diameter (*P* = 0.0043), and morphological grade of the inner cell mass and trophectoderm (*P* < 0.0001 and *P* < 0.0001, respectively), were significantly different between the groups. After propensity score matching, the clinical records of 1,910 patients in each group were retrospectively analysed; the characteristics of patients and transferred blastocysts were comparable between the groups (Table 1).

**Table 1:** Patient and cycle characteristics

	Before propensity score matching			After propensity score matching		
	Natural	Letrozole	*P*-value	Natural	Letrozole	*P*-value
No. of patients, *n*	12,700	1,911		1,910	1,910	
Female age (years), (range)	38.7 ± 0.0 ^a^ (23–49)	36.7 ± 0.1 ^b^ (22–46)	< 0.0001	36.7 ± 0.1 (26–50)	36.7 ± 0.1 (26–49)	0.8844
Male age (years), (range)	40.1 ± 0.0^a^ (25–69)	38.4 ± 0.1 ^b^ (26–68)	< 0.0001	38.4 ± 0.1 (25–62)	38.4 ± 0.1 (26–68)	0.5857
Body mass index	20.8 ± 0.0	20.9 ± 0.1	0.1204	20.9 ± 0.1	20.9 ± 0.1	0.8924
Serum AMH level	2.1 ± 0.0	2.9 ± 0.2	< 0.0001	2.9 ± 0.2	2.9 ± 0.2	0.5464
Previous OR cycles	1.7 ± 0.0	1.7 ± 0.0	0.8249	1.7 ± 0.0	1.7 ± 0.0	0.1817
Previous ET cycles	0.6 ± 0.0	0.6 ± 0.0	0.0891	0.6 ± 0.0	0.6 ± 0.0	0.1704
Previous deliveries	4,024 (31.7)	816 (42.7)	< 0.0001	838 (43.9)	816 (42.7)	0.4725
Previous miscarriages	3,363 (26.5)	456 (23.9)	0.0151	436 (22.8)	456 (23.9)	0.4443
Previous caesarean section	887 (7.0)	111 (5.8)	0.0579	135 (7.1)	111 (5.8)	0.1137
Previous smoking	812 (6.4)	127 (6.7)	0.6753	137 (7.2)	127 (6.7)	0.5235
Insemination methods						0.9196
cIVF, *n* (%)	4,836 (38.1)	694 (36.3)	0.1386	691 (36.2)	694 (36.3)	
ICSI, *n* (%)	7,864 (61.9)	1,217 (63.7)		1,219 (63.8)	1,216 (63.7)	
Culture time to the expanded blastocyst stage (h)	126.7 ± 0.1	125.4 ± 0.3	< 0.0001	125.4 ± 0.3	125.4 ± 0.3	0.9238
Blastocyst diameter (μm)	173.3 ± 0.4	176.3 ± 0.9	0.0043	180.4 ± 0.5	180.2 ± 0.5	0.1250
Morphological grade						
ICM Grade A, *n* (%)	3,373 (26.6)	588 (30.8)	< 0.0001	573 (30.0)	587 (30.7)	0.6913
Grade B, *n* (%)	4,827 (38.0)	763 (39.9)		753 (39.4)	763 (40.0)	
Grade C, *n* (%)	4,500 (35.4)	560 (29.3)		584 (30.6)	560 (29.3)	
TE Grade A, *n* (%)	2,967 (23.4)	552 (28.9)	< 0.0001	534 (28.0)	552 (28.9)	0.7410
Grade B, *n* (%)	4,054 (31.9)	619 (32.4)		615 (32.2)	618 (32.4)	
Grade C, *n* (%)	5,678 (44.7)	740 (38.7)		761 (39.8)	740 (38.7)	

The data are shown as means and standard errors of the mean, unless otherwise indicated

*AMH* anti-müllerian hormone, *OR* oocyte retrieval, *ET* embryo transfer, *cIVF* conventional in vitro fertilization, *ICSI* intracytoplasmic sperm injection, *ICM* inner cell mass, *TE* trophectoderm

### Pregnancy outcomes after SVBTs in natural and letrozole cycles

The serum levels of oestradiol, progesterone, and luteinising hormone on the day of the trigger were significantly lower in the letrozole than in the natural group (*P* < 0.0001, *P* = 0.0003, and *P* < 0.0001, respectively; Table 2). The serum oestradiol level on the day of SVBT was significantly lower in the letrozole than in the natural group (*P* < 0.0001; Table 2). The ratio of patients with an insufficient luteal function who were vaginally administered progesterone was significantly lower in the letrozole than in the natural group (Table 2). Conversely, the serum progesterone level was significantly higher in the letrozole group (*P* < 0.0001). The endometrial thickness on the day of SVBT was comparable between the groups. Although no difference was observed regarding the implantation rates between the groups, in the letrozole group, the rates of clinical pregnancy, ongoing pregnancy, and live birth were significantly higher than those in the natural group (*P* = 0.0180, *P* = 0.0212, *P* = 0.0411, respectively; Table 2). The incidence of early pregnancy loss was significantly lower in the letrozole group (*P* = 0.0014). The incidence of miscarriage, twin pregnancy, and stillbirth was comparable between the groups. Furthermore, the analysed cohort was stratified by patient age (young patients, < 37 years; advanced age patients, ≥ 37 years). In the young patients, although the implantation rates were comparable between the groups, the early pregnancy loss rate was significantly lower in the letrozole group than in the natural group, resulting in higher clinical and ongoing pregnancy rates and tendency of improvement in live birth in the letrozole group (Supplementary Table 3). Although the decreased early pregnancy loss was observed in the letrozole group, the clinical and ongoing pregnancies and live birth were comparable between the groups in patients with advanced age.

**Table 2:** Pregnancy outcomes after SVBT in natural and letrozole cycles

	Natural	Letrozole	*P*-value
ET cycles, *n*	1,910	1,910	
Oestradiol on the day of the trigger (pg/mL)	335.7 ± 3.5	258.5 ± 3.0	< 0.0001
Progesterone on the day of the trigger (ng/mL)	0.5 ± 0.0	0.4 ± 0.0	0.0003
Luteinising hormone on the day of the trigger (mIU/mL)	18.0 ± 0.2	14.1 ± 0.2	< 0.0001
Oestradiol on the day of SVBT (pg/mL)	169.5 ± 1.7	114.2 ± 1.5	< 0.0001
Progesterone on the day of SVBT (ng/mL)	16.6 ± 0.1	22.0 ± 0.2	< 0.0001
Insufficient luteal function, *n* (%)	258 (13.5)	84 (4.4)	< 0.0001
Endometrial thickness on the day of SVBT (mm)	10.6 ± 0.0	10.7 ± 0.0	0.0273
Implantation, *n* (%)	1,088 (57.0)	1,120 (58.6)	0.2945
Clinical pregnancies, *n* (%)	962 (50.4)	1,035 (54.2)	0.0180
Ongoing pregnancies, *n* (%)	850 (44.5)	921 (48.2)	0.0212
Live birth, *n* (%)	746 (39.1)	808 (42.3)	0.0411
Early pregnancy loss, *n* (%)	126 (11.6)	85 (7.8)	0.0014
Miscarriages, *n* (%)	214 (22.3)	226 (21.8)	0.8254
Twin pregnancies, *n* (%)	10 (1.3)	10 (1.2)	0.8599
Still birth, *n* (%)	2 (0.3)	1 (0.1)	0.5181

The data are shown as means and standard errors of the mean, unless otherwise indicated

SVBT, single vitrified-warmed blastocyst transfer

Univariate logistic analysis revealed significant associations between the live birth rate, ages of male and female participants, body mass index, embryo culture time, blastocyst diameter, blastocyst morphology, and endometrial preparation method (Table 3). Multivariate logistic regression analysis was also performed to adjust for potential statistical co-founding biases (Table 3). The endometrial preparation method was significantly associated with the live birth rate, even after adjustment for confounders; the administration of letrozole in the SVBT cycle significantly improved the live birth rate (AOR, 1.156 [95% CI, 1.006–1.329]; *P* = 0.0397).

**Table 3:** Univariate and multivariate logistic regression analyses of live birth after single vitrified-warmed blastocyst transfer

		Univariate analysis				Multivariate analysis			
		Odds ratio	95% CI	*P*-value	AUC	Adjusted odds ratio	95% CI	*P*-value	AUC
Female age (years)		0.876	0.861–0.982	< 0.0001	0.642	0.888	0.868–0.909	< 0.0001	0.711
Male age (years)		0.939	0.92–0.952	< 0.0001	0.592	0.996	0.979–1.013	0.6814	
Body mass index		0.970	0.947–0.993	0.0128	0.523	0.988	0.979–1.013	0.3616	
Culture time to the expanded blastocyst stage		0.962	0.956–0.967	< 0.0001	0.621	0.975	0.968–0.982	< 0.0001	
Blastocyst diameter		1.008	1.005–1.011	< 0.0001	0.561	1.007	1.004–1.011	< 0.0001	
Gardner’s criteria									
ICM	Grade A	Reference	-	-	0.615	Reference	-	-	
	Grade B	0.729	0.625–0.850	< 0.0001		1.097	0.919–1.309	0.3015	
	Grade C	0.315	0.264–0.376	< 0.0001		0.746	0.591–0.941	0.0135	
TE	Grade A	Reference	-	-	0.640	Reference	-	-	
	Grade B	0.586	0.497–0.691	< 0.0001		0.702	0.585–0.843	0.0002	
	Grade C	0.273	0.231–0.322	< 0.0001		0.492	0.394–0.615	< 0.0001	
Endometrial preparation									
Natural		Reference	-	-		Reference	-	-	
Letrozole		1.144	1.005–1.301	0.0412	0.516	1.156	1.006–1.329	0.0397	

*ET* embryo transfer, *ICM* inner cell mass, *TE* trophectoderm, *CI* confidence interval, *AUC* area under the curve

### Perinatal outcomes after SVBTs in natural and letrozole-induced cycles

The perinatal outcomes in the live-birth cycles were stratified according to the endometrial preparation (Table 4). Female age, male age, and body mass index were comparable between the groups. Furthermore, the incidence rate of pregnancy complications and the caesarean section rate were comparable between the groups. Gestational age, birth length, birth weight, infant sex, and incidence of birth defects were statistically comparable between the groups. Table 5 shows the multivariate logistic regression analysis of maternal and perinatal outcomes after SVBTs, including a comparison of the letrozole-induced and natural cycles. Univariate logistic regression analysis was performed, and confounders were identified (Supplemental Table 3). No adverse effects of letrozole-induced endometrial preparation on maternal and perinatal outcomes were observed.

**Table 4:** Neonatal outcomes in live birth cycles stratified by the ovarian stimulation method

	Natural	Letrozole	*P*-value
Live birth, *n*	746	808	
Female age	35.5 ± 0.1	35.6 ± 0.1	0.6882
Male age	37.8 ± 0.2	37.5 ± 0.2	0.2048
Body mass index	20.6 ± 0.1	20.8 ± 0.1	0.1734
Pregnancy complications, *n* (%)	37 (5.0)	52 (6.4)	0.2110
Hypertensive disorders of pregnancy, *n* (%)	8 (1.1)	3 (0.4)	0.0996
Gestational diabetes mellitus, *n* (%)	4 (0.5)	7 (0.9)	0.4380
HELLP syndrome, *n* (%)	0 (0)	1 (0.1)	0.3365
Preterm premature rupture of membrane, *n* (%)	1 (0.1)	4 (0.5)	0.2093
Low-lying placenta, *n* (%)	4 (0.5)	4 (0.5)	0.9099
Placenta previa, *n* (%)	9 (1.2)	10 (1.2)	0.9554
Placental abruption, *n* (%)	1 (0.1)	2 (0.3)	0.6107
Others, *n* (%)	1 (0.1)	2 (0.3)	0.6107
Caesarean section rate, *n* (%)	220 (29.5)	216 (26.7)	0.2267
Gestational age, weeks	39.0 ± 0.1	39.0 ± 0.1	0.6539
Preterm delivery (< 37 weeks), *n* (%)	40 (5.4)	45 (5.6)	0.8575
Birth length (cm)	49.1 ± 0.1	49.0 ± 0.1	0.7309
Birth weight (g)	3,031 ± 16	3,017 ± 15	0.5208
Low birth weight (< 2,500 g), *n* (%)	66 (8.9)	72 (8.9)	0.9648
Small for gestational age	31 (4.2)	34 (4.2)	0.9589
Large for gestational age	126 (16.9)	116 (14.4)	0.1688
Infant sex			0.9515
Male, *n* (%)	388 (52.0)	419 (51.9)	
Female, *n* (%)	358 (48.0)	389 (48.1)	
Birth defect, *n* (%)	31 (4.2)	36 (4.5)	0.7755

The data are shown as means and standard errors of the mean, unless otherwise indicated

**Table 5:** Multivariate logistic regression analysis of neonatal outcomes after single vitrified-warmed blastocyst transfer in the letrozole-induced cycle

Outcomes	Adjusted odds ratio	95% CI	*P*-value
Pregnancy complications ^a^	1.304	0.844–2.015	0.2313
Hypertensive disorders of pregnancy ^a^	0.341	0.089–1.304	0.1162
Gestational diabetes mellitus ^a^	1.403	0.400–4.910	0.5960
Preterm premature rupture of membrane ^a^	3.314	0.366–29.984	0.2863
Low-lying placenta ^a^	0.885	0.219–3.568	0.8642
Placenta previa ^a^	1.036	0.418–2.565	0.9383
Placental abruption ^a^	1.857	0.167–20.557	0.6137
Caesarean section ^b^	0.884	0.690–1.133	0.3321
Preterm delivery (< 37 weeks) ^c^	1.058	0.669–1.676	0.8069
Low birth weight (< 2,500 g) ^d^	1.023	0.711–1.473	0.8995
Small for gestational age ^e^	1.026	0.623–1.689	0.9178
Large for gestational age ^f^	0.800	0.606–1.056	0.1163
Birth defect ^g^	1.061	0.649–1.735	0.8113

*CI* confidence interval

Reference: Natural group

^a^ Confounders: body mass index and twin pregnancy. ^b^ Confounders: female age, male age, body mass index, previous caesarean section, and twin pregnancy. ^c^ Confounders: twin pregnancy. ^d^ Confounders: female age, body mass index, and twin pregnancy. ^e^ Confounders: body mass index. ^f^ Confounders: body mass index, developmental speed, morphological grade of inner cell mass, and twin pregnancy. ^g^ Confounders: morphological grade of inner cell mass

### Detailed analysis of the congenital anomalies

Congenital anomalies were categorised into nine types, as shown in Supplementary Table 4. The incidence of each congenital anomaly was similar between the two groups. The most frequent congenital anomaly was circulatory defects in both groups.

## Discussion

Although letrozole-induced endometrial preparation has recently attracted much attention, the advantages of letrozole administration on pregnancy outcomes after FBT, when compared with the natural cycle FBTs, remain unknown. Our study provided initial evidence that letrozole-induced endometrial preparation improved the live birth rate after SVBT when compared with natural cycles in ovulatory patients. Furthermore, this is an index report demonstrating that letrozole-induced endometrial preparation did not increase the incidence of pregnancy complications and congenital anomalies after SVBTs when compared to natural cycles.

Propensity score matching was performed in the present study as the patient characteristics and transferred blastocyst quality were significantly different between the natural and letrozole groups, before matching. After matching, the baseline characteristics of the two cohorts were comparable; subsequently, the cycle characteristics and pregnancy outcomes were compared between the two groups. We first demonstrated the similarity in the endometrial thickness between the natural and letrozole groups, which confirmed the previous findings [19, 37]. Our results also demonstrated that in the letrozole group, the rates of clinical pregnancy, ongoing pregnancy, and live birth after SVBTs were significantly improved by decreasing the early pregnancy loss, compared with those in the natural group. Progesterone plays key roles in embryo implantation as well as maintenance of early pregnancy; insufficient luteal function is one of the reasons for early pregnancy loss and miscarriages in the first trimester [38, 39]; therefore, the decrease of early pregnancy loss and improvement of live birth after letrozole administration might be associated with the increase of luteal function. Our recent study reported that ovarian stimulation with letrozole slightly increased the number of growing follicles compared with the natural cycle (1.3 vs. 1.1) [40], which might lead to the increased number of corpus lutea, increased serum progesterone level, and subsequent improvement of the live birth rate in the letrozole group. Furthermore, functional improvement of the corpus luteum may be another reason for the higher observed live birth rate after letrozole-induced endometrial preparation. In the letrozole group, the serum progesterone level on the SVBT day significantly increased, as compared with that in the natural group. Letrozole administration suppresses the conversion of androgen into oestradiol, resulting in an increased androgen concentration in granulosa cells. Androgen reportedly promotes the expression of follicular-stimulating hormone (FSH) receptor in granulosa cells [41]. FSH stimulates progesterone secretion in mural granulosa cells, which differentiate into luteal cells after ovulation [42]. This suggests that letrozole administration promotes progesterone synthesis in luteal granulosa cells via accumulated androgen-induced FSH receptor upregulation. Further molecular studies are required to reveal the mechanism by which letrozole administration increases the serum progesterone level on the day of transfer. A previous study reported that the use of the natural cycles for timing FETs should be reserved for younger patients, probably because the luteal phase defects are more common in patients with advanced maternal age [43]. Thus, letrozole was expected to have a potential of improving luteal function and outcome in patients with advanced maternal age. Contrary to our expectation, the live birth rate was not improved in patients with advanced age although an improvement in the luteal function was observed, suggesting that the pregnancy outcomes in patients with advanced age were more affected by other factors, such as abnormal chromosomal status and low developmental competence.

Recently, several studies have found fewer complications after FBTs in natural cycles compared with FBTs in artificial (hormone replacement) cycles [44–48]. An aberrant oestradiol level and insufficient luteal function increased the risks of adverse maternal and neonatal outcomes [47, 49–51]. Therefore, it is suggested that endometrial preparation during the natural cycle is the preferred treatment in women with ovulatory cycles undergoing FBT when an increased risk of obstetrical complications and potential neonatal complications after FBTs with artificial cycles is expected. In the present study, we observed no adverse effects from letrozole administration on maternal and neonatal outcomes after SVBTs. Letrozole administration resulted in a low level of oestradiol and stimulated luteal function; thus, letrozole-induced endometrial preparation did not increase both pregnancy complications and congenital anomalies after SVBTs compared with natural cycles. Furthermore, the letrozole-induced endometrial preparation did not affect the incidence of twin pregnancy; this result validate the previous finding [19].

The strength of the present study was the examination of a large dataset obtained from a single centre. Moreover, the use of letrozole, techniques of ET, and culture conditions were uniform in this study. Furthermore, in the present study, propensity score matching was performed to balance baseline characteristics between the natural and letrozole groups. Therefore, a consideration of bias due to differences regarding various potential detailed conditions was not necessary. Moreover, this study included patient treatment history, embryonic development speed, and blastocyst morphological grade as confounders, as these were significantly associated with the live birth rate after SVBTs when the multivariate logistic regression analysis was performed.

Nevertheless, this study had certain limitations; our findings were not compared with the incidence of pregnancy complications and congenital anomalies in natural pregnancy. Furthermore, this study was retrospective in nature; thus, further multicentre studies are required to ascertain the generalisability of these findings to other clinics with different protocols and/or patient demographics. Even though we used propensity score matching and multivariate models to adjust for confounders, we can never adjust for all possible confounders; therefore, there is a possibility that the results of the present study might be biased by the better prognosis of patients in the letrozole group. Data were collected using self-reported parental questionnaires on maternal and neonatal outcomes. Self-reported maternal and neonatal complications could be potentially erroneous, particularly in cases where uncommon/complex medical terms were involved. Furthermore, we conducted power analysis between the natural and letrozole groups and detected a power of 98.1% for the incidence of live birth. However, this study detected a power of 71.6% for pregnancy complications and a power of 9.1% for birth defects between the groups; therefore, the accuracy of some results was low because of the small sample size, especially for the rare events, including the birth defects (i.e. the incidence rates of birth defects in the natural and letrozole groups were 4.2% and 4.5%, respectively). To detect a 0.3% increase in the birth defect with a power of 80%, at least 35,000 live birth cycles (22 times larger cohort than that of our study) should be included. Therefore, further randomised controlled trials with larger sample sizes are required to validate our findings.

## Conclusions

In conclusion, our results indicated that letrozole-induced endometrial preparation improved the live birth rate without adverse effects on perinatal outcomes and congenital anomalies after FBT cycles. Furthermore, letrozole administration increased the serum progesterone level on the day of transfer; therefore, letrozole-induced endometrial preparation might be more effective in patients with insufficient luteal function. Further clinical studies are required to determine which patients will benefit more from letrozole-induced endometrial preparation.

## Supplementary Information

**Additional file 1: Supplementary Figure 1. **Flowchart illustrating the method of patient selection, including the inclusion and exclusion criteria.

**Additional file 2.**

**Additional file 3: Supplementary Table 1.** *P*-values of univariate logistic regression analysis between confounders and outcomes **Supplementary Table 2**. Embryonic outcomes stratified by the ovarian stimulation method **Supplementary Table 3.** Pregnancy outcomes after SVBT in natural and letrozole cycles, stratified by the female age **Supplementary Table 4.** Congenital anomalies stratified by the ovarian stimulation method.

## Data Availability

The datasets used and/or analysed during the current study available from the corresponding author on reasonable request.

## Abbreviations

AOR: Adjusted odds ratio
CC: Clomiphene citrate
CI: Confidence interval
ET: Embryo transfers
FBT: Frozen blastocyst transfers
FET: Frozen embryo transfer
FSH: Follicular-stimulating hormone
LGA: Large for gestational age
OR: Odds ratio
SGA: Small for gestational age
SVBT: Single vitrified-warmed blastocyst transfer
