# Supplementary material for: Letrozole-induced endometrial preparation improved the pregnancy outcomes after frozen blastocyst transfer compared to the natural cycle: a retrospective cohort study
Source: BMC Pregnancy Childbirth. 2022 Nov 7;22:824. doi: 10.1186/s12884-022-05174-0 (PMC9639274; doi:10.1186/s12884-022-05174-0)
Supplement: Supplementary file 1 — Additional file 1: Supplementary Figure 1. Flowchart illustrating the method of patient selection, including the inclusion and exclusion criteria. [file 12884_2022_5174_MOESM1_ESM.docx]

**Figure caption**

**Supplementary Figure 1.** Flowchart illustrating the method of patient selection, including the inclusion and exclusion criteria
