# Supplementary figures and images for: Letrozole-induced endometrial preparation improved the pregnancy outcomes after frozen blastocyst transfer compared to the natural cycle: a retrospective cohort study
Source: BMC Pregnancy Childbirth. 2022 Nov 7;22:824. doi: 10.1186/s12884-022-05174-0 (PMC9639274; doi:10.1186/s12884-022-05174-0)

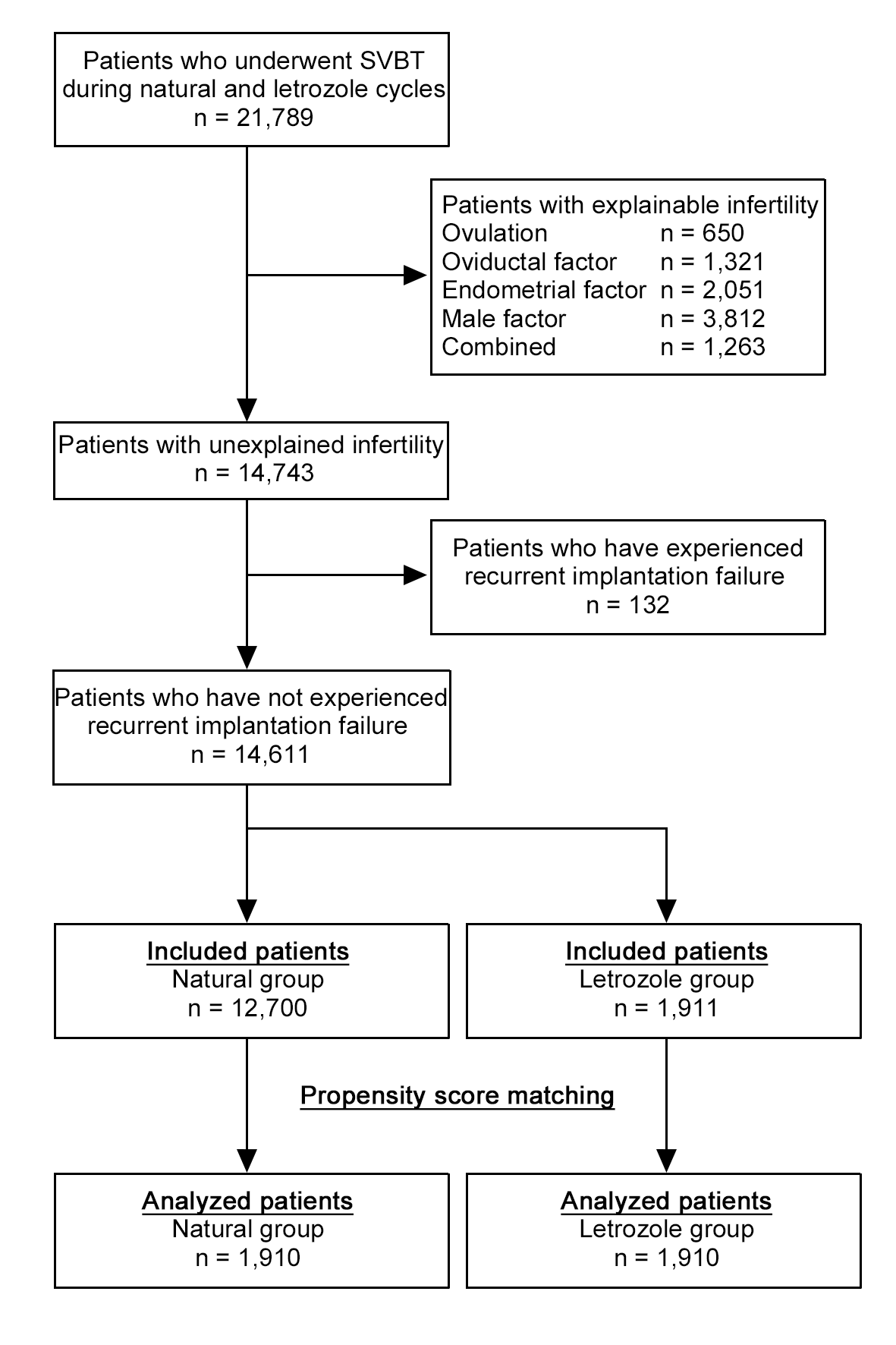

Supplement: Supplementary file 2 — Additional file 2. [file 12884_2022_5174_MOESM2_ESM.tif]
