# Supplementary material for: Letrozole-induced endometrial preparation improved the pregnancy outcomes after frozen blastocyst transfer compared to the natural cycle: a retrospective cohort study
Source: BMC Pregnancy Childbirth. 2022 Nov 7;22:824. doi: 10.1186/s12884-022-05174-0 (PMC9639274; doi:10.1186/s12884-022-05174-0)
Supplement: Supplementary file 3 — Additional file 3: Supplementary Table 1. P-values of univariate logistic regression analysis between confounders and outcomes Supplementary Table 2. Embryonic outcomes stratified by the ovarian stimulation method Supplementary Table 3. Pregnancy outcomes after SVBT in natural and letrozole cycles, stratified by the female age Supplementary Table 4. Congenital anomalies stratified by the ovarian stimulation method. [file 12884_2022_5174_MOESM3_ESM.docx]

Supplementary Table 1. *P*-values of univariate logistic regression analysis between confounders and outcomes

|  | Female age | Male age | Body mass index | Previous caesarean section | Developmental speed | Inner cell mass | Twin pregnancy |
| --- | --- | --- | --- | --- | --- | --- | --- |
| Pregnancy complications | 0.1153 | 0.7466 | 0.0731 | 0.9366 | 0.3815 | 0.4858 | 0.0724 |
| Caesarean section | < 0.0001 | 0.0131 | 0.0009 | < 0.0001 | 0.9045 | 0.1683 | < 0.0001 |
| Preterm delivery (< 37 weeks) | 0.1813 | 0.5700 | 0.9030 | 0.6909 | 0.8925 | 0.8056 | < 0.0001 |
| Low birth weight (< 2,500 g) | 0.0791 | 0.9580 | 0.0291 | 0.8859 | 0.3572 | 0.5926 | < 0.0001 |
| Small for gestational age | 0.5233 | 0.4177 | 0.0374 | 0.7966 | 0.6767 | 0.8342 | 0.8542 |
| Large for gestational age | 0.7882 | 0.5841 | 0.0015 | 0.1244 | 0.0359 | 0.0304 | 0.0532 |
| Birth defect | 0.8278 | 0.7462 | 0.1852 | 0.1321 | 0.2098 | 0.0881 | 0.8792 |

Supplementary Table 2. Embryonic outcomes stratified by the ovarian stimulation method

|  | Natural | Letrozole | *P*-value |
| --- | --- | --- | --- |
| No. of oocyte retrieval cycles | 1,910 | 1,910 |  |
| No. of matured oocytes | 2.3 ± 0.0 | 2.3 ± 0.0 | 0.2293 |
| No. of oocytes fertilised | 2.1 ± 0.0 | 2.1 ± 0.0 | 0.2011 |
| Fertilisation rate (%) | 89.9 ± 0.3 | 90.1 ± 0.3 | 0.3036 |
| No. of cleaved embryos | 2.0 ± 0.0 | 2.0 ± 0.0 | 0.2010 |
| Cleavage rate (%) | 89.7 ± 0.3 | 89.9 ± 0.3 | 0.2890 |
| No. of blastocysts | 1.6 ± 0.0 | 1.6 ± 0.0 | 0.2586 |
| Blastocyst formation (%) | 78.1 ± 0.5 | 77.6 ± 0.6 | 0.6197 |
| No. of blastocysts cryopreserved | 1.5 ± 0.0 | 1.5 ± 0.0 | 0.2591 |
| Blastocyst cryopreservation rate (%) | 74.0 ± 0.5 | 74.2 ± 0.5 | 0.8727 |

The data are presented as means and standard errors of the mean.

Supplementary Table 3. Pregnancy outcomes after SVBT in natural and letrozole cycles, stratified by the female age

|  | Female age < 37 years | | |  | Female age ≥ 37 years | | |
| --- | --- | --- | --- | --- | --- | --- | --- |
|  | Natural | Letrozole | *P*-value |  | Natural | Letrozole | *P*-value |
| ET cycles, *n* | 875 | 917 |  |  | 1,035 | 993 |  |
| Oestradiol on the day of SVBT (pg/mL) | 173.8 ± 2.6 | 116.6 ± 2.2 | <0.0001 |  | 166.0 ± 2.3 | 111.8 ± 2.0 | <0.0001 |
| Progesterone on the day of SVBT (ng/mL) | 16.8 ± 0.2 | 22.1 ± 0.2 | <0.0001 |  | 16.5 ± 0.1 | 21.9 ± 0.2 | <0.0001 |
| Endometrial thickness on the day of SVBT (mm) | 10.8 ± 0.0 | 10.9 ± 0.0 | 0.0429 |  | 10.4 ± 0.1 | 10.6 ± 0.1 | 0.0414 |
| Implantation, *n* (%) | 567 (64.8) | 618 (67.4) | 0.2462 |  | 521 (50.3) | 502 (50.6) | 0.9226 |
| Clinical pregnancies, *n* (%) | 510 (58.3) | 577 (62.9) | 0.0446 |  | 452 (43.7) | 458 (46.1) | 0.2672 |
| Ongoing pregnancies, *n* (%) | 464 (53.0) | 532 (58.0) | 0.0337 |  | 386 (37.3) | 389 (39.2) | 0.3839 |
| Live birth, *n* (%) | 428 (48.9) | 487 (53.1) | 0.0759 |  | 318 (30.7) | 321 (32.3) | 0.4377 |
| Early pregnancy loss, *n* (%) | 57 (10.1) | 41 (6.6) | 0.0328 |  | 69 (13.2) | 44 (8.8) | 0.0223 |
| Miscarriages, *n* (%) | 80 (15.7) | 89 (15.4) | 0.9054 |  | 134 (29.7) | 137 (29.9) | 0.9299 |
| Twin pregnancies, *n* (%) | 7 (1.6) | 5 (1.0) | 0.4219 |  | 3 (0.9) | 5 (1.6) | 0.4850 |
| Still birth, *n* (%) | 2 (0.5) | 1 (0.2) | 0.4906 |  | 0 (0) | 0 (0) | – |

Supplementary Table 4. Congenital anomalies stratified by the ovarian stimulation method

|  | Natural | Letrozole | *P*-value |
| --- | --- | --- | --- |
| Live birth, *n* | 746 | 808 |  |
| Chromosomal abnormalities, *n* (%) | 5 (0.7) | 5 (0.6) | 0.8992 |
| Circulatory, *n* (%) | 8 (1.1) | 10 (1.2) | 0.7610 |
| Nervous system, *n* (%) | 3 (0.4) | 0 (0) | 0.0712 |
| Digestive systems, *n* (%) | 1 (0.1) | 1 (0.1) | 0.9549 |
| Urogenital, *n* (%) | 1 (0.1) | 2 (0.3) | 0.6107 |
| Musculoskeletal, *n* (%) | 3 (0.4) | 8 (1.0) | 0.1672 |
| Respiratory, *n* (%) | 2 (0.3) | 2 (0.3) | 0.9363 |
| Reproductive organ, *n* (%) | 4 (0.5) | 5 (0.6) | 0.8302 |
| Other congenital abnormalities, *n* (%) | 2 (0.3) | 1 (0.1) | 0.5172 |
| Unknown malformation, *n* (%) | 3 (0.4) | 2 (0.3) | 0.5908 |
